# Sexual Dimorphism of Dexamethasone as a Prophylactic Treatment in Pathologies Associated With Acute Hypobaric Hypoxia Exposure

**DOI:** 10.3389/fphar.2022.873867

**Published:** 2022-05-20

**Authors:** Neha Chanana, Tsering Palmo, Kavita Sharma, Rahul Kumar, Bhushan Shah, Sudhanshu Mahajan, Girish M. Palleda, Mohit D. Gupta, Ritushree Kukreti, Mohammad Faruq, Tashi Thinlas, Brian B. Graham, Qadar Pasha

**Affiliations:** ^1^ Department of Genomics and Molecular Medicine, CSIR-Institute of Genomics and Integrative Biology, Delhi, India; ^2^ Department of Medicine, University of California, San Francisco, San Francisco, CA, United States; ^3^ Department of Cardiology, GB Pant Institute of Post Graduate Medical Education and Research, New Delhi, India; ^4^ Department of Medicine, Sonam Norboo Memorial Hospital, Leh, Ladakh, India; ^5^ Institute of Hypoxia Research, New Delhi, India

**Keywords:** high-altitude, acute mountain sickness, pulmonary hypertension, dexamethasone, sexual dimorphism

## Abstract

Dexamethasone can be taken prophylactically to prevent hypobaric hypoxia-associated disorders of high-altitude. While dexamethasone-mediated protection against high-altitude disorders has been clinically evaluated, detailed sex-based mechanistic insights have not been explored. As part of our India-Leh-Dexamethasone-expedition-2020 (INDEX 2020) programme, we examined the phenotype of control (*n* = 14) and dexamethasone (*n* = 13) groups, which were airlifted from Delhi (∼225 m elevation) to Leh, Ladakh (∼3,500 m), India, for 3 days. Dexamethasone 4 mg twice daily significantly attenuated the rise in blood pressure, heart rate, pulmonary pressure, and drop in SaO_2_ resulting from high-altitude exposure compared to control-treated subjects. Of note, the effect of dexamethasone was substantially greater in women than in men, in whom the drug had relatively little effect. Thus, for the first time, this study shows a sex-biased regulation by dexamethasone of physiologic parameters resulting from the hypoxic environment of high-altitude, which impacts the development of high-altitude pulmonary hypertension and acute mountain sickness. Future studies of cellular contributions toward sex-specific regulation may provide further insights and preventive measures in managing sex-specific, high-altitude–related disorders.

## Introduction

Exposure to high-altitude (HA, ≥2500 m) can cause HA illnesses, including acute mountain sickness (AMS), high-altitude pulmonary edema (HAPE), pulmonary hypertension (PH), and high-altitude cerebral edema (HACE). The mechanism of these disorders is complex, involving multiple clinical symptoms and biological pathways (Beall, 2003). Oxygen-sensing is central among the contributory pathways. Various markers in these pathways contribute to the genotype to phenotype response, thereby shaping the adaptation or maladaptation to hypobaric hypoxic environments (Qadar Pasha et al., 2001; Bigham et al., 2013; Mishra et al., 2015a).

Pulmonary hypertension (PH) is characterized by vascular remodeling caused by abnormal smooth muscle production and increased pulmonary arteriolar resistance, depleted bioavailability of vasodilators such as nitric oxide (NO), and enhanced vasoconstrictors (Naeije, 2010; Chanana et al., 2020). PH can result from acute hypoxia and chronic exposure to high-altitudes. Of interest, there is a phenotype of sexual dimorphism in PH; women are known to be more predisposed to PH than men, but men have worse outcomes after developing PH (Mair et al., 2014; Martin and Pabelick, 2014). While female sex hormones and their metabolites are detrimental to the development of PH, the influence of sex hormones on the underlying pathophysiology remains unanswered, and data are conflicting (Dempsie and MacLean, 2013).

Dexamethasone, a corticosteroid, is commonly prescribed to individuals upon induction to altitude or prophylactically prior to ascending to HA (Maggiorini, 2010; Subudhi et al., 2011). Dexamethasone increases oxyhemoglobin saturation and reduces the hypoxia-induced rise in pulmonary arterial pressure in HAPE-sensitive individuals (Ferrazzini et al., 1987; Maggiorini et al., 2006; Fischler et al., 2009). Furthermore, it stimulates ventilatory acclimatization to hypoxia, thereby ameliorating the symptoms of acute mountain sickness (Liu et al., 2013). Mechanistically, dexamethasone inhibits hypoxia-induced pulmonary endothelial dysfunction and controls the HA-induced increase in pulmonary arterial pressure by stimulating cGMP production, which activates nitric oxide synthase and increases sympathetic activity to increase oxygen uptake (Maggiorini et al., 2006; Maggiorini, 2010). Furthermore, dexamethasone reduces the permeability of cells and the capillary wall, thereby reducing the leakage of pulmonary fluid and the associated symptoms of edema (Swenson, 2016). It is relevant to add that both sexes travel to HA equally; however, little is known about differences in the severity of HA disorders between the two sexes. Both men and women are susceptible, but whether one sex is more vulnerable due to initial clinical differences that contribute to the physiological function is unclear. A meta-analysis study based on 18 eligible prospective studies concluded that women have a higher prevalence of AMS (Hou et al., 2019). Dexamethasone is known for sex-specific clinico-physiological actions regarding inflammatory diseases (Duma et al., 2010; Kroon et al., 2020). However, a sex-specific regulation by dexamethasone in the hypoxic environment of HA has not been explored. Hence, in our India-Leh-Dexamethasone-expedition-2020 (INDEX 2020) study, we aimed to determine the sex-based efficacy of dexamethasone prophylaxis in relation to clinical symptoms and associated PH and AMS in lowlanders traveling to HA. We hypothesized that compared to men, the dexamethasone prophylaxis would offer greater protection to women from developing AMS or high-altitude–related complications.

## Materials and Methods

### Study Design and Participants

The INDEX 2020 study was conducted between 26th September 2020 and 1st October 2020, starting at the lowland in the Govind Ballabh (GB) Pant Hospital, Delhi (∼225 m), India, and traveling to the Sonam Norboo Memorial (SNM) Hospital, Leh, Ladakh (∼3,500 m), India, to evaluate the efficacy of dexamethasone in preventing altitude-induced clinical changes in a sex-specific manner**.** Participants provided written informed consent, and the protocol was approved by the human ethical committees of the Council of Scientific and Industrial Research-Institute of Genomics and Integrative Biology, Delhi, India, and the SNM Hospital, Leh, Ladakh, India. All procedures were performed in compliance with relevant laws and institutional guidelines.

A total of 27 healthy lowland volunteers of both genders aged 24–28 years participated in the study. The volunteers were randomly divided into two groups: control (Ctrl, *n* = 14) and dexamethasone (Dex, *n* = 13). For sex-based studies, the ctrl group (*n* = 14) had six women and eight men, and the ex group (*n* = 13) had six women and seven men. Subjects with chronic diseases, pulmonary infection, pregnant women, or those unable to give informed consent or who did not comply with the study protocol were excluded.

### Time-Frame of the Experimental Procedure and Biomedical Assessment

After undergoing baseline clinical, hematological, blood biochemistry, radiological, and echocardiographic evaluations at the GB Pant Hospital, Delhi (low altitude, LA), on day 0, the subjects were airlifted to Leh, Ladakh (high-altitude, HA), for 3 days at 3500 m. The flight took one and a half hours to reach Leh. Dexamethasone (4 mg twice a day) (Wockhardt Ltd., India) (Ellsworth et al., 1987) was orally administered 24 h prior to induction to HA and continued for the next 3 days during the stay at HA (4 days total) under the supervision of clinical investigators. The treatment was unblinded; control subjects received no medication. Clinical parameters including systolic and diastolic blood pressure (BP), heart rate (HR), arterial oxygen saturation (SaO_2_), and Lake Louise Acute Mountain Sickness (AMS) Score were conducted at LA and then evaluated every 24 h for 3 days at HA, while radiological and echocardiographic evaluations were also assessed on day 3 at HA. Furthermore, echocardiography was additionally re-evaluated at LA, 7 days after returning from HA, to ascertain the return of the clinico-physiological state of the volunteers. Sample collection, protocol procedure, and biomedical assessment are presented in Supplementary Table S1 in chronological order.

### Assessment of Clinical Parameters

On day 0, the medical history was recorded, and the blood examination, including hemogram and routine biochemistry, was performed. All participants underwent anthropometric measurements, including height, weight, and body mass index (BMI). Furthermore, the clinical examination included measurement of HR, SBP, and DBP in the supine position after 15 min of rest every 24 h for 2 days at LA, prior to travel, and for 3 days at HA by automatic digital blood pressure monitor (Omron HEM 7120, Japan). SaO_2_ was measured at LA prior to travel to HA and twice daily at HA using finger-pulse oximetry (Omron CMS50N Contec, Japan).

### Lake Louise Acute Mountain Sickness Score

The Lake Louise Acute Mountain Sickness Score was evaluated every 24 h for 3 days at HA. The score consists of four symptoms (headache, nausea/vomiting, fatigue, and dizziness/light-headedness), each on a scale of 0–3, and a total score ≥3 including at least one point for headache was considered diagnostic for AMS (Roach et al., 2018).

### Chest X-Ray

Chest radiographs were obtained using X-ray machines at LA and on day 3 of HA (Siemens, Germany at LA and Allengers X-ray, India at HA) in order to determine the high-altitude pulmonary edema (HAPE).

### Transthoracic Echocardiography

Echocardiographic examination was performed by a qualified and experienced cardiologist blinded to treatment assignment using Epiq-7 (Philips Medical Systems, Andover, MA, United States) at GB Pant Hospital, Delhi (LA), and eSAOTE—MyLabAlpha, (eSAOTE, United States) at SNM Hospital, Leh (HA). TTE was assessed thrice: once at baseline LA before the initiation of treatment and prior to travel to HA, on the 3rd day at HA, and again at LA 7 days after returning from HA.

The left and right heart chamber dimensions were determined according to the American Society of Echocardiography (ASE) recommendations (Lang et al., 2015). The ejection fraction was calculated by the summation of disc method (biplane Simpson’s rule) from the apical two- and four-chamber view (Lang et al., 2015). In the apical four-chamber view, tricuspid annular plane systolic excursion (TAPSE) was obtained by M-mode to assess RV systolic function. Pulmonary arterial systolic pressure (PASP) was calculated using continuous wave (CW) Doppler. A coaxial tricuspid regurgitant (TR) jet was identified in the parasternal long-axis (RV inflow), parasternal short axis, or apical four-chamber view with the help of color Doppler. CW Doppler was used to achieve a satisfactory envelope. The peak TR jet velocity of the envelope was then measured. PASP was calculated by the modified Bernoulli equation [PASP = 4 V^2^ + mean right atrial pressure (RAP)]. Mean right atrial pressure (RAP) was estimated from inferior vena cava (IVC) size and collapsibility using ASE recommendations (Rudski et al., 2010). In the absence of right ventricular outflow obstruction, PASP (pulmonary arterial systolic pressure) is equal to RVSP (right ventricular systolic pressure).

### Statistical Analysis

Data are presented as means and standard errors of the mean (SEMs, represented by error bars in histograms). Comparisons of the difference in the mean of two groups (±SEM) were carried out using one-way ANOVA and the two-tailed unpaired Student’s t-test. All statistical tests were carried out using Sigma Plot, version 12. *p* < 0.05 was considered statistically significant. The changes in clinical parameters for each group upon induction to HA are presented as the differences between average values of the respective parameters for 3 days at HA and the value at day 0 at LA. Comparisons between the intervention and control groups were made by comparing respective changes upon induction to HA to those of LA.

## Results

### Baseline Clinical Characteristics, Hematocrit, and Routine Blood Chemistry at LA

Prior to HA travel, the baseline clinical characteristics and hematocrit profile were similar among participants in the two groups, that is, the Ctrl and the Dex groups **(** *p* = ns for all categories, Supplementary Table S2).

### Dexamethasone Prevented Acute Mountain Sickness at High-Altitude in a Sex-Specific Manner

The Lake Louise Score (LLS) for the two groups for 3 days at HA is shown in Table 1. On day 1 at HA, three control subjects out of 14 (21%) had a total score ≥3, including at least one score due to headache in the setting of an ascent altitude. In the dexamethasone group, three subjects out of 13 (23%) had scores ≥3. On subsequent days at HA, the number of subjects with elevated LLS decreased in the control group, and no subject with AMS was seen in the dexamethasone group (Table 1).

**TABLE 1:** Lake Louise Score in the control and dexamethasone groups at HA.

	Day 1 at HA		Day 2 at HA		Day 3 at HA	
	AMS	No AMS	AMS	No AMS	AMS	No AMS
Control group (*n* = 14, 6F+8M)						
Number	3 (21.4%)	11	2 (14.3%)	12	1 (7.1%)	13
Females	2F (33.3%)	4F	2F (33.3%)	4F	1F (16.7%)	5F
Males	1M (12.5%)	7M	0M (0%)	8M	0M (0%)	8M
Mean ± SD	4.0 ± 1.4	1.5 ± 1.2	3.5 ± 0.5	1.0 ± 0.9	3.0 ± 0.0	0.8 ± 1.1
Dexamethasone group (*n* = 13, 6F+7M)						
Number	3 (23.1%)	10	0	13	0	13
Females	2F (33.3%)	4F	0F	6F	0F	6F
Males	1M (14.3%)	6M	0M	7M	0M	7M
Mean ± SD	4.0 ± 0.0	1.1 ± 1		0.8 ± 0.8	0	1 ± 1.2

Data are presented as mean ± SEM. AMS, acute mountain sickness; n, number of subjects.

#### Sex-Based Differentiation

Interestingly, two out of six women (33.3%) had LLS ≥3 on initial exposure to HA in each of the control and dexamethasone groups. On subsequent days at HA, the number of female subjects with elevated LLS completely resolved in the dexamethasone group but persisted in the control group. On the other hand, the male subjects displayed similar AMS trends in both the control and dexamethasone groups (Table 1). Therefore, other clinical parameters were evaluated, emphasizing sex-specific patterns between dexamethasone and control groups.

### Dexamethasone Attenuated Blood Pressure Elevation With Greater Protection in Women at High-Altitude

**FIGURE 1:**
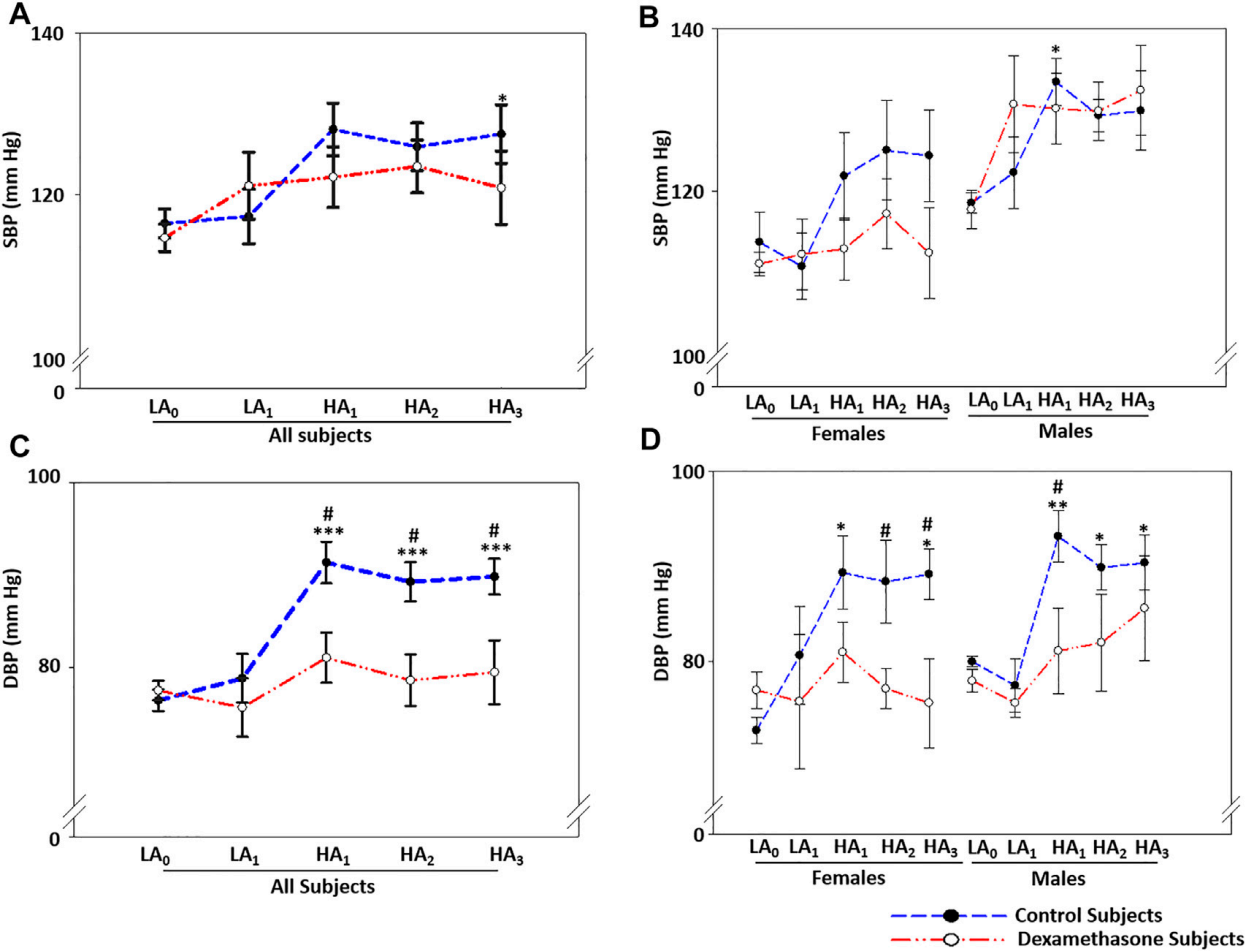
Dexamethasone attenuated BP elevation at HA with a greater protection to women. **(A)** Dexamethasone attenuated SBP-elevation by ∼3.2 mmHg for 3 days at HA compared to the elevation in the control groups. **(B)** Dexamethasone attenuated SBP-elevation in women by ∼7.0 mmHg, while it did not control SBP in men. **(C)** Dexamethasone attenuated DBP-elevation by ∼10.8 mmHg when compared to the elevation in the control groups. **(D)** Dexamethasone controlled DBP-elevation at HA in women by ∼15.4 mmHg and in men by ∼6.9 mmHg compared to elevations in the respective control groups. Data are presented as mean ± SE and are compared by one-way ANOVA. */^#^ *p* < 0.05, **/^##^ *p* < 0.01, and ***/^###^ *p* < 0.001 were considered statistically significant. * represents significance within each group compared to respective control at day 0 at LA, while ^#^ represents significance between the control group and dexamethasone group for the respective time point. LA_0_, day 0 at low altitude; LA_1_, day 1 at low altitude; HA_1_, day 1 at high-altitude; HA_2_, day 2 at high-altitude; HA_3_, day 3 at high-altitude.

The two groups at LA had normal SBP that elevated upon induction to HA and remained elevated during the 3 days of stay **(** Figure 1A, Supplementary Table S3A). For the 3 days at HA, the SBP increased by a mean of 10.7 mmHg (*p* = 0.012) and 7.5 mmHg (*p* = ns) in the control and the dexamethasone groups, respectively, compared to the SBP of the respective group at LA (Supplementary Table S3A). Thus, dexamethasone relatively attenuated the SBP rise by ∼3.2 mmHg (Supplementary Table S3A).

#### Sex-Based Differentiation

Notably, the increase in SBP and its interaction with dexamethasone were substantially sex-biased (Figure 1B, Supplementary Table S3B). The female control group had a mean SBP elevation of ∼10.1 mmHg at HA compared to LA, while the female dexamethasone group had a mean elevation of only 3.1 mmHg with a protective effect of ∼7 mmHg **(** *p* = ns, Supplementary Table S3B pink). In contrast, the males in the control group had a mean SBP elevation of 11.4 mmHg at HA compared to the same at LA (*p* = 0.030), and the male dexamethasone group had a rise of 12.1 mmHg under similar comparisons (Figure 1B, Supplementary Table S3B blue).

The two groups had normal DBP at LA, which increased upon induction to HA and remained elevated during the 3 days of stay **(** Figure 1C, Supplementary Table S4A). For the 3 days at HA, the DBP was elevated by a mean of ∼13.3 mmHg in the control group (*p* ≤ 0.001), while the DBP increased by only 2.5 mmHg in the dexamethasone group (*p* = ns) (Supplementary Table S4A). Dexamethasone relatively attenuated the DBP rise by a mean of ∼10.8 mmHg (*p* < 0.05) against the elevation in the control group (Supplementary Table S4A).

#### Sex-Based Differentiation

The DBP trend was similar to SBP for the total period of the experiment and was sex-biased (Figure 1D, Supplementary Table S4B). The female control group had an elevation of 16.3 mmHg in DBP **(** *p* ≤ 0.05, Supplementary Table S4B pink). Of note, however, DBP in women who received dexamethasone only increased by 0.9 mmHg (*p* = ns) (Figure 1D, Supplementary Table S4B pink), showing an effective protection of 15.4 mmHg (*p* < 0.05) (Figure 1B, Supplementary Table S4B Pink). In comparison, the male control group had an elevation of ∼11.1 mmHg (*p* < 0.01, Figure 1D, Supplementary Table S4B blue), and the male dexamethasone group had a DBP elevation of 4.2 mmHg (*p* = ns) (Figure 1B), showing effective protections of 6.8 mmHg (*p* < 0.05); thus, dexamethasone was significantly more effective at controlling the DBP rise (Figure 1D, Supplementary Table S4B blue).

### Dexamethasone-Mediated Heart Rate Control Was More Evident in Women at High-Altitude

**FIGURE 2:**
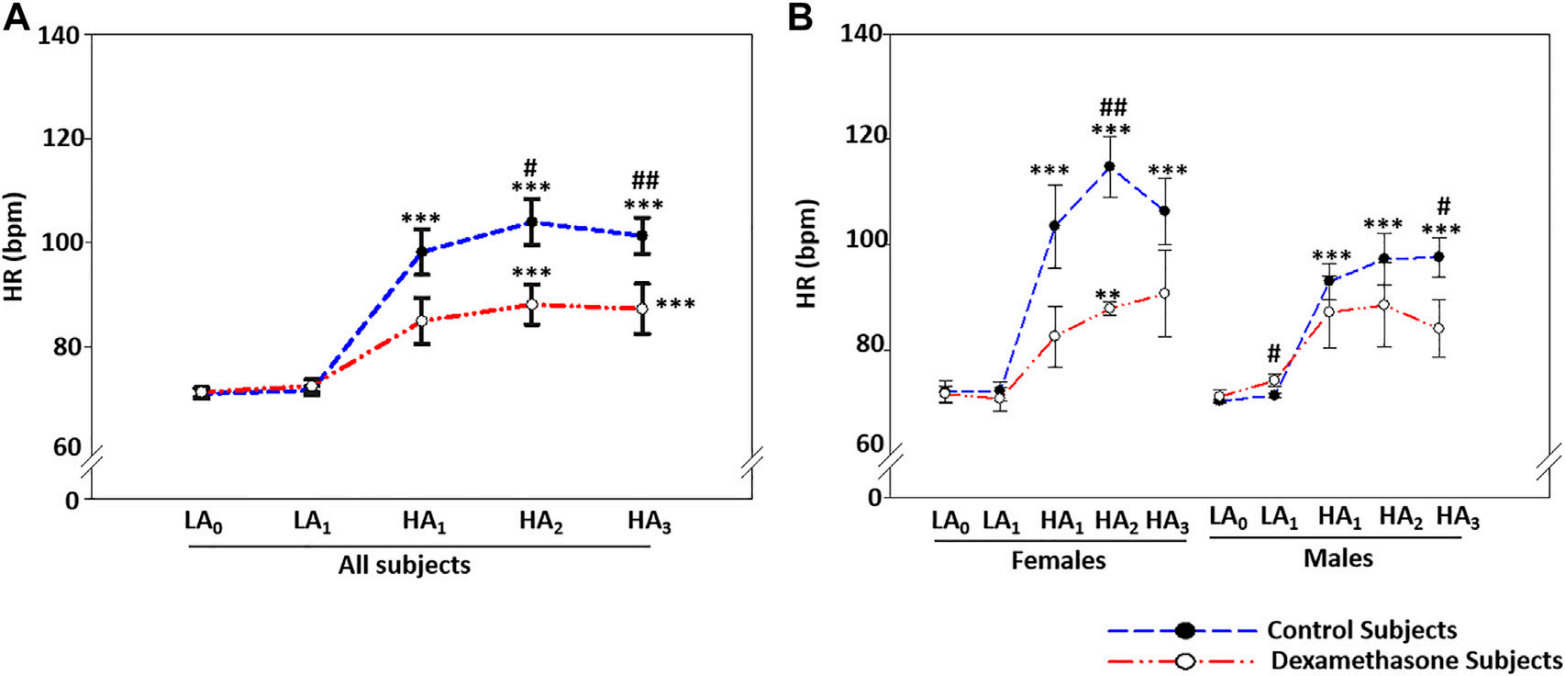
Dexamethasone mediated-HR control at HA was more evident in women. **(A)** Dexamethasone controlled HR-elevation at HA by ∼14.7 bpm compared to controls. **(B)** Dexamethasone controlled HR-elevation at HA in women by ∼20.9 bpm and in men by ∼10.3 bpm compared to elevation in the respective control groups. Data are presented as mean ± SE and are compared by one-way ANOVA. */^#^ *p* < 0.05, **/^##^ *p* < 0.01, and ***/^###^ *p* < 0.001 were considered statistically significant. * represents significance within each group compared to respective control at day 0 at LA, while ^#^ represents significance between the control group and dexamethasone group for the respective time point. HA_1_, day 1 at high-altitude; HA_2_, day 2 at high-altitude; HA_3_, day 3 at high-altitude.

The two groups had an average heart rate (HR) of ∼71 beats/min (bpm) at LA, but it elevated significantly upon induction to HA and remained elevated for the 3 days of stay (Figure 2A, Supplementary Table S5A). For the 3 days at HA, HR increased by a mean of ∼30.1 bpm in controls (*p* ≤ 0.001) and 15.4 bpm in the dexamethasone group (*p* ≤ 0.001; Figure 2A, Supplementary Table S5A). Dexamethasone controlled the HR efficiently at HA, with the HR attenuation being ∼50% (*p* < 0.05, Supplementary Table S5A).

#### Sex-Based Differentiation

Furthermore, sex-based differences in HR were seen at HA (Figure 2B). At HA, the HR increased by ∼36.2 bpm in the female control group and ∼25.7 bpm in the male control group (*p* ≤ 0.001, Figure 2B, Supplementary Table S5B). The HA-induced HR elevation in the dexamethasone-treated groups was ∼15.3 bpm (*p* = 0.005) in women and ∼15.4 bpm (*p* = ns) in men (Figure 2B), showing a protective effect of 20.9 bpm in women (*p* = 0.004) versus ∼10.3 bpm in men (*p* = 0.043) against the respective controls (Figure 2B, Supplementary Table S5B).

### Dexamethasone Attenuated Pulmonary Pressures at High-Altitude More Predominantly in Women

Echocardiography-based heart function parameters differed in the two groups at HA (Table 2, Supplementary Table S6).

**TABLE 2:** Echocardiogram parameters, PASP, RAP, and pulmonary hypertension at LA and HA in the control and the dexamethasone groups.

Parameters	Time-point	Control	*p*-value	Dexamethasone	*p*-value
		group (*n* = 14)		group (*n* = 13)	
A. PASP		mmHg		mmHg	
All subjects	LA (Pre-induction to HA)	19.0 ± 1.1	-	17.0 ± 1.2	-
	HA	35.7 ± 4.4	*	30.6 ± 3.3	**
	LA (Upon return from HA)	21.1 ± 1.8		20.7 ± 2.4	
Females	LA (Pre-induction to HA)	20.0 ± 1.4	-	17.3 ± 1.4	-
	HA	42.7 ± 7.3	ns	30.6 ± 4.2	ns
	LA (Upon return from HA)	22.4 ± 2.7		20.0 ± 2.4	
Males	LA (Pre-induction to HA)	17.7 ± 1.5	-	16.7 ± 1.9	-
	HA	29.7 ± 4.0	ns	30.7 ± 4.9	ns
	LA (Upon return from HA)	19.8 ± 2.1		21.3 ± 3.5	
B. RAP		mmHg		mmHg	
All subjects	LA (Pre-induction to HA)	4.4 ± 0.0	-	5.0 ± 0.0	-
	HA	7.1 ± 0.6	*p* = 0.007	6.5 ± 0.6	*p* = 0.033
	LA (Upon return from HA)	5.0 ± 0.0		5.0 ± 0.0	
Females	LA (Pre-induction to HA)	5.0 ± 0.0	-	5.0 ± 0.0	-
	HA	8.3 ± 1.0	*p* = 0.065	6.7 ± 1.0	ns
	LA (Upon return from HA)	5.0 ± 0.0		5.0 ± 0.0	
Males	LA (Pre-induction to HA)	5.0 ± 0.0	-	5.0 ± 0.0	-
	HA	6.3 ± 0.8	ns	6.4 ± 0.8	ns
	LA (Upon return from HA)	5.0 ± 0.0		5.0 ± 0.0	
C. PH					
All subjects	Total PH	6/14 = 42.8%		4/13 = 30.8%	
	Mild PH (PASP: 35–50 mmHg)	4		4	
	Moderate PH (PASP: 50–70 mmHg)	1		0	
	Severe PH (PASP: >70 mmHg)	1		0	
Females	Total PH	4/6 = 66.7%		1/6 = 16.7%	
	Mild PH	2		1	
	Moderate PH	1		0	
	Severe PH	1		0	
Males	Total PH	2/8 = 25%		2/7 = 28.6%	

*p < 0.05 was considered statistically significant; ns, nonsignificant.

Data are presented as mean ± SEM.; n, number of subjects; PASP, pulmonary arterial systolic pressure; RAP, right atrial pressure; PH, pulmonary hypertension; LA, low altitude; HA, high-altitude

**FIGURE 3:**
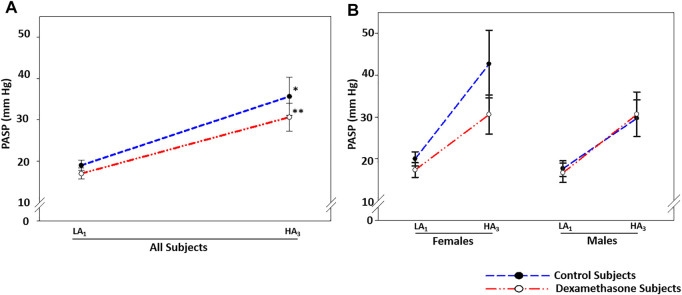
Dexamethasone-mediated PASP-control at HA was predominant in women. **(A)** Dexamethasone attenuated the PASP elevation by ∼3.1 mmHg. **(B)** Dexamethasone attenuated PASP by ∼9.5 mmHg in women, while it could not control the rise in PASP in men. Data are presented as mean ± SE and are compared by one-way ANOVA. *p < 0.05, **p < 0.01, and ***p < 0.001 were considered statistically significant. * represents significance within each group compared to respective control. LA_1_, day 1 at low altitude; HA_1_, day 1 at high-altitude; HA_3_, day 3 at high-altitude.

At HA, PASP elevated by approximately 16.7 and 13.6 mmHg in control (*p* = 0.023) and dexamethasone (*p* = 0.010) groups, respectively (Figure 3A, Table 2A). Dexamethasone attenuated the PASP elevation by ∼3.1 mmHg. PASP returned to near pre-induction levels in both groups upon returning to LA.

#### Sex-Based Differentiation

In women, PASP increased by 22.7 mmHg in the control group (*p* = 0.067) and by ∼13.3 mmHg in the dexamethasone group (*p* = 0.082) (Figure 3B, Table 2A pink), with an attenuation of ∼9.5 mmHg. Induction of men to HA caused almost similar mean elevations in PASP of 12.0 and 14.0 mmHg in control and dexamethasone groups, respectively **(** *p* = ns, Figure 3B and Table 2A blue). Thus, dexamethasone could protect against the HA-induced rise in PASP in women but not in men.

The IVC diameter for both the sexes at LA and HA are provided in Supplementary Table S6. Upon induction to HA, the IVC diameter increased by 0.8 and 0.6 cm in the control (*p* ≤ 0.001) and dexamethasone (*p* ≤ 0.001) groups, respectively (Supplementary Table S6A). Upon returning to LA, the IVC size was restored to near normal in all the groups. In addition, at HA, the IVC was collapsible (IVC collapsibility >50%) in eight control subjects out of 14 and in nine dexamethasone subjects out of 13 (Supplementary Table S6B). The calculated RAP increased by an average of 2.7 and 1.5 mmHg in the control and dexamethasone groups (*p* = 0.007 and *p* = 0.033), respectively (Table 2C). Thus, dexamethasone attenuated the HA-induced RAP elevation by 1.2 mmHg compared to the elevation in the control group.

#### Sex-Based Differentiation

In women at HA compared to those at LA, the IVC diameter increased by 0.7 and 0.6 cm in the control (*p* = 0.006) and dexamethasone (*p* = 0.003) groups, respectively (Supplementary Table S6A pink). In addition, the IVC was collapsible (IVC collapsibility >50%) in two control subjects out of six and in four dexamethasone subjects out of six (Supplementary Table S6B pink). Based on changes in the IVC size and collapsibility, RAP was increased by ∼3.3 and ∼1.7 mmHg in female control (*p* = 0.065) and dexamethasone (*p* = ns) groups, respectively (Table 2C pink). In men at HA compared to those at LA, the IVC diameter increased by 0.8 and 0.7 cm in the control (*p* ≤ 0.001) and dexamethasone (*p* = 0.001), groups, respectively (Supplementary Table S6A blue). In addition, the IVC was collapsible for six control subjects out of eight and five dexamethasone subjects out of seven (Supplementary Table S6B blue). Based on changes in the IVC size and collapsibility of men, the RAP increased by approximately 1.3 and 1.4 mmHg in the control and dexamethasone groups, respectively (*p* = ns, Table 2C blue), indicating that dexamethasone was comparatively less effective in men.

PH was calculated based on the PASP values at HA (Table 2C). It was concluded that six control subjects out of 14 (42.8%) displayed PH (Table 2C). Of these six subjects, four had mild PH (PASP: 35–50 mmHg), one had moderate PH (PASP: 50–70 mmHg), and one had severe PH (PASP: >70 mmHg). On the other hand, four dexamethasone subjects out of 13 (30.8%) displayed mild PH (Table 2C).

#### Sex-Based Differentiation

In women, four control subjects out of six (66.7%) displayed PH at HA (Table 2C pink). Of these, two had mild, one had moderate, and one had severe PH. On the other hand, one of six dexamethasone subjects (16.7%) showed only mild PH (PASP: 35–50 mmHg) at HA (Table 2C pink). In the case of men, two control subjects out of eight (25%) displayed mild PH (PASP: 35–50 mm) (Table 2C blue), whereas two dexamethasone-treated male subjects out of seven (28.6%) displayed mild PH (PASP: 35–50 mmHg) (Table 2C blue). Thus, we observed a superior influence of dexamethasone in controlling the PH in women at HA.

Other echocardiography parameters including left ventricle and right ventricle function, left ventricle size, left auricle and right auricle size, interventricular septum dimension in end-diastole, and posterior wall in the end-diastole did not change upon induction to HA in the two groups (Supplementary Table S7).

### Dexamethasone-Mediated SaO_2_ Control Was Similar in Both Sexes

**FIGURE 4:**
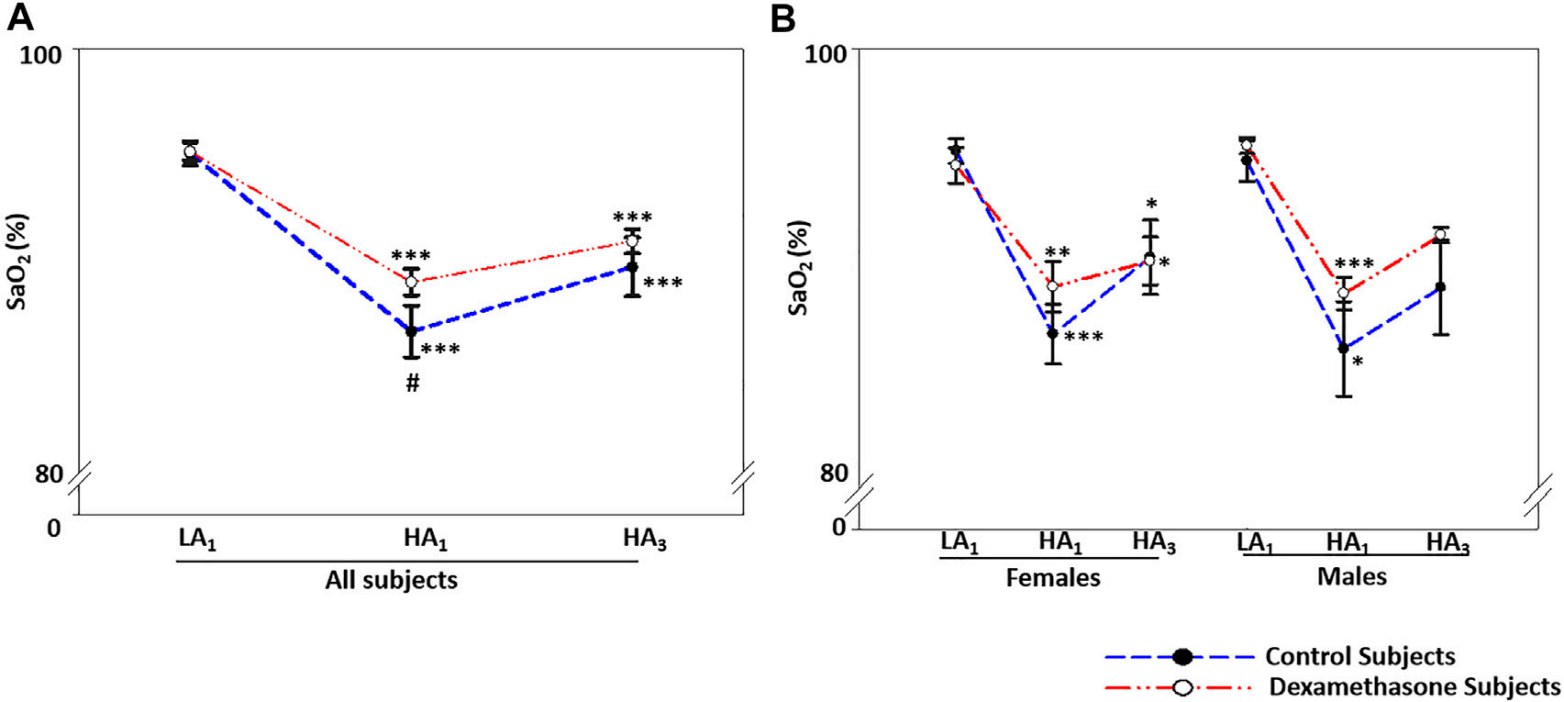
Dexamethasone-mediated SaO2-control at HA was similar in both the sexes. **(A)** Dexamethasone ameliorated SaO_2_ by ∼1.7% compared to controls. **(B)** Dexamethasone showed a similar control of SaO_2_ in both the sexes, that is, by ∼1.7% in women and ∼1.8% in men. Data are presented as mean ± SE and are compared by one-way ANOVA. *p < 0.05, **p < 0.01, and ***p < 0.001 were considered statistically significant. * represents significance within each group compared to respective control. LA_1_, day 1 at low altitude; HA_1_, day 1 at high-altitude; HA_3_, day 3 at high-altitude.

SaO_2_ was normal at LA for the two groups; upon induction to HA and subsequent 3 days’ stay, it decreased by approximately 6.9% (*p* < 0.001) and 5.2% (*p* < 0.001) in the control and dexamethasone groups, respectively (Figure 4A, Supplementary Table S8A); a protection of 1.7% (*p* = ns) (Figure 4A).

#### Sex-Based Differentiation

SaO_2_ at HA was similar between the two sexes (Figure 4B). On moving from LA to HA, SaO_2_ decreased by ∼6.6% (*p* < 0.01) and ∼7.2% (*p* < 0.05) (Figure 4B, Supplementary Table S8B) in the female and male control groups, respectively. The respective decrease in SaO_2_ was ∼4.9% (*p* < 0.05) and ∼5.4% (*p* ≤ 0.001) in the dexamethasone female and male groups (Figure 4B, Supplementary Table S8B). Thus, the effect of dexamethasone was approximately 1.7% in women and 1.8% in men (Figure 4B).

### Chest X-Rays Were Normal for Both the Groups at High-Altitude

Chest X-ray including bilateral lung fields, bilateral hila, bilateral CP (costophrenic) angle, cardiac shadow, and bony and soft tissue was normal in all participants in the two groups at both LA and HA (data not shown).

## Discussion

In this study, we analyzed high-altitude–associated clinical and cardiac changes, along with susceptibility toward PH, AMS, or HAPE with and without dexamethasone in young, healthy volunteers of both sexes traveling to HA. A significant finding was sexual dimorphism in response to dexamethasone, with a more pronounced protective effect observed in women than in men. In general, dexamethasone appeared effective at blocking the effects of high elevation, except the decrease in SaO_2_ at HA.

We observed elevated BP, HR, and pulmonary pressure and depleted SaO_2_ levels in the subjects ascending from LA to HA. These observations align with the available reports (Bärtsch, and Gibbs, 2007; Parati et al., 2013). The increase in BP and HR is likely associated with the hypoxia-mediated increased sympathetic activity (Bärtsch and Gibbs, 2007; Parati et al., 2013), resulting in greater cardiac contractility and heart rate, coupled with increased constriction of peripheral blood vessels. Increased PASP is clinically correlated with several diseases including PH and heart failure (Bursi et al., 2012; Maron et al., 2018). Hypoxia-induced vascular remodeling is associated with several physiological processes including potassium and calcium channel activities, reduced vasodilators such as nitric oxide, and increased vasoconstrictors such as endothelin, thromboxane A2, and angiotensin-converting enzyme 1 (Ali et al., 2012; Dunham-Snary et al., 2017). PH is known to occur in several high-altitude illnesses and is a key feature of HAPE (Maggiorini and Leon-Velarde, 2003). In this study, we observed AMS in a few of the subjects, but no cases of HAPE.

A striking finding was that the high-altitude–associated clinical changes were sex-biased. Sex differences occur in the regulation of BP; men having higher SBP and HR than women (Boos et al., 2017; Horiuchi et al., 2019). Testosterone contributes to BP *via* the renin–angiotensinogen aldosterone system and oxidative stress (Reckelhoff, 2001). In this study, men had greater increases in SBP, and women had greater increases in DBP and HR at HA. These data are in agreement with previous studies (Reckelhoff, 2001; Boos et al., 2017; Horiuchi et al., 2019) looking at the perturbation of signaling pathways at HA, including vascular, anti-diuretic, and vascular growth factors (Mishra et al., 2015b; Richalet, 2016; Chanana et al., 2020). The HA-associated increase in pulmonary pressure was comparatively more in women than in men. A recent study showed that women were more likely to have higher PASP than men and were more predisposed to heart failure (Lakshmanan et al., 2020). Furthermore, this study aligns with the increased incidence of PH in women than men, where endogenous sex hormones, especially 17β-estradiol and its metabolites, play a role in developing the disease (White et al., 2011; Mair et al., 2014).

Another important respiratory parameter at HA is SaO_2_, which is known to decrease upon induction to HA (Beall, 2003). In this study, the fall in SaO_2_ was comparatively greater in men than in women, which aligns with previous studies (Bhaumik et al., 2008; Nishimura et al., 2020). The lower levels of SaO_2_ tend to alter several hypoxia-sensing genes, such as *HIF-1α*, *HIF-2α*, *EGLN1*, and others (Mishra et al., 2013). Differential regulation of these genes contributing to varied regulation of several dependent markers can alter physiological functions (Petousi and Robbins, 2014).

Dexamethasone is prescribed to check AMS symptoms at HA, either preventatively or by reversal (Subudhi et al., 2011). In this study, prophylactic treatment with dexamethasone effectively controlled high-altitude–associated clinical changes at HA. Dexamethasone reduces PAP, raises oxygen saturation, and has been reported to suppress NFĸB-mediated inflammation, thereby decreasing hypoxia-induced PH in HA sojourners (O'Hara et al., 2014; Price et al., 2015). Furthermore, there is a possibility that dexamethasone blocks Rho kinase mediated acute vasoconstriction directly through unknown mechanisms or indirectly by blocking the recruitment of inflammatory immune cells in hypoxia (Kumar et al., 2020). In addition, increased apical alveolar membrane Na^+^ channels, basal Na^+^K^+^-ATPase, stimulated surfactant secretion, and protein exudate prevention may add to dexamethasone-mediated protection (Guney et al., 2007).

Here, we found that the effect of dexamethasone prophylaxis on HA-induced pathophysiology was sex-biased; these observations are the first of their kind. Dexamethasone could be an effective treatment to control the BP, HR, and PASP; these changes were more apparent in women than in men. Consequently, dexamethasone provided greater protection to women by lowering their susceptibility toward PH and AMS. Such a female-oriented protective action of dexamethasone at HA could be related to its greater transcriptional regulation of hypoxia signaling pathways (Duma et al., 2010), differences in the epigenetics present in women, or other female-specific hormonal differences. Moreover, glucocorticoid receptor–mediated gender-specific regulation of inflammatory gene expression could also regulate the observed gender-specific dexamethasone responses (Quinn and Cidlowski, 2016). Furthermore, glucocorticoid crosstalk with sex hormones, described for metabolic disease, cancer, and inflammation (Kroon et al., 2020), could contribute to sexual dimorphism. However, the underlying mechanisms need further validation.

**FIGURE 5:**
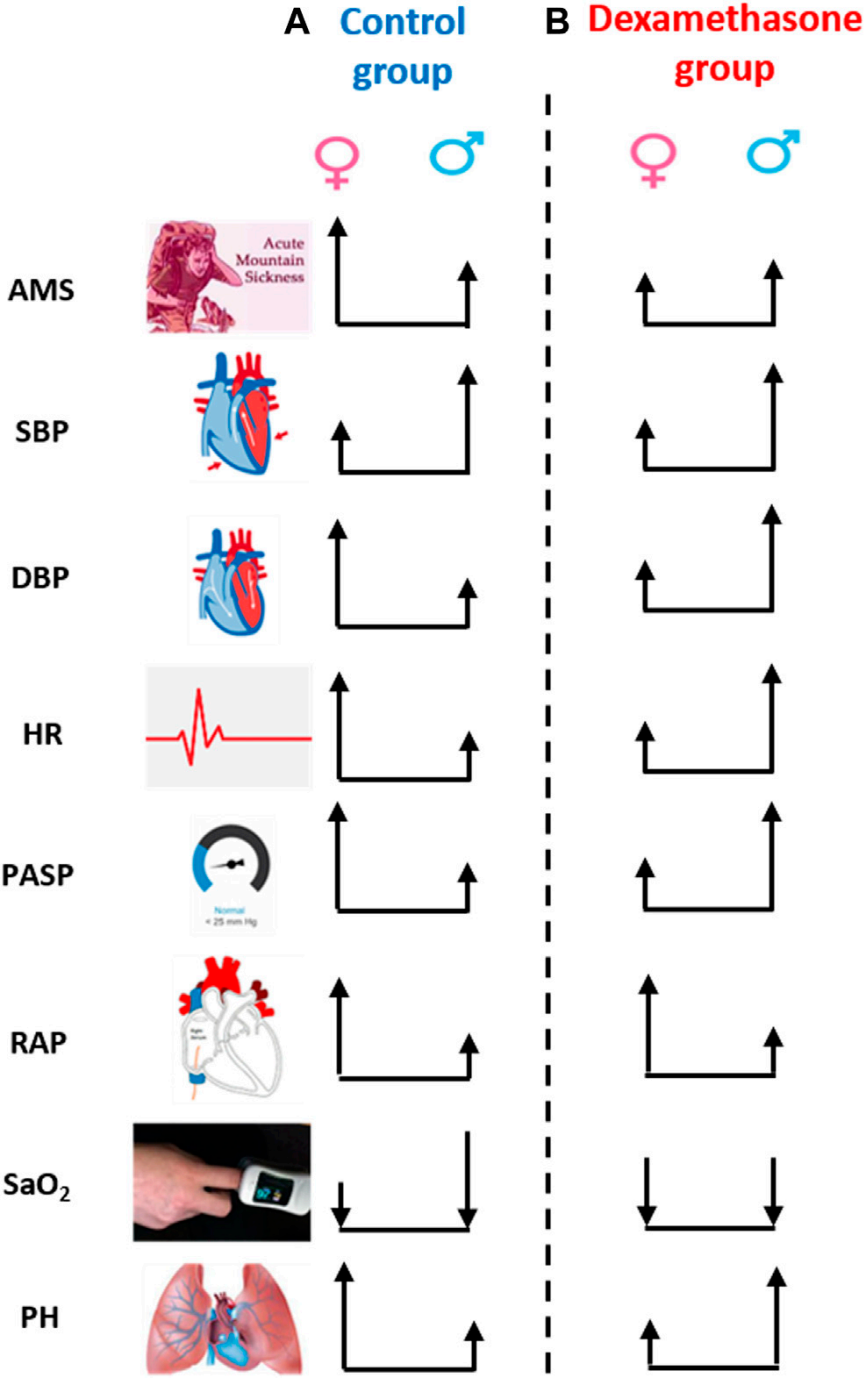
Sex-biased clinical regulation by dexamethasone at high-altitude. The height of arrow represents the numerical change in the respective parameter on moving from low-altitude to high-altitude, and the upside and downside direction represents the increase or decrease, respectively. **(A)** Change in parameters, except for SBP and SaO_2_, was more evident in women than in men in the control group, that is, women were more susceptible to high-altitude. **(B)** However, the change in parameter, except for RAP, was less in women than in men in the dexamethasone group. Dexamethasone controlled DBP, HR, and PASP more effectively and prevented AMS and PH at HA in women. AMS, acute mountain sickness; SBP, systolic blood pressure; DBP, diastolic blood pressure; HR, heart rate; PASP, pulmonary arterial systolic pressure; RAP, right atrial pressure; SaO_2_, arterial oxygenation; PH, pulmonary hypertension.

In conclusion, this study identified sex-specific clinical changes upon induction to HA (Figure 5); women were more vulnerable to AMS and PH at HA but dexamethasone prophylaxis effectively controlled such changes in women. This novel finding opens avenues to explore the cellular and molecular mechanistic insights underlying such a sex-specific regulation by dexamethasone at high-altitude. Our study underscores sex to be considered a key biological variable in the design and interpretation of clinical studies. Further validation and mechanistic studies may substantiate the current findings, and the clinicians may consider these data as to how best to approach the prophylactic treatment of high-altitude travelers.

### Limitation to the Study

Our study with a subject size of 27 provided a precise sex-biased regulation in physiologic parameters by dexamethasone under the high-altitude hypoxic environment. Nonetheless, further validation is needed in a larger sample size of male/female subgroups and/or in animal models. Transporting a larger number of humans to high-altitudes in the two groups could be challenging. In addition, we did not identify HAPE by X-ray findings in this group; hence, the effectiveness of dexamethasone in HAPE prevention could not be investigated and correlated with other parameters. An increased sympathetic tone can raise RVSP/mPAP *via* either increased cardiac output and/or increased pulmonary vascular resistance (PVR); thus, echocardiographic stroke volume (SV) and cardiac output (CO) estimation can be included in future investigations.

## Data Availability

The original contributions presented in the study are included in the article/Supplementary Material, further inquiries can be directed to the corresponding author.
