# Supplementary material for: Sexual Dimorphism of Dexamethasone as a Prophylactic Treatment in Pathologies Associated With Acute Hypobaric Hypoxia Exposure
Source: Front Pharmacol. 2022 May 20;13:873867. doi: 10.3389/fphar.2022.873867 (PMC9163683; doi:10.3389/fphar.2022.873867)
Supplement: Supplementary file 1 [file DataSheet1.pdf]

Supplementary Tables

Table S1: Sample collection in chronological order

Steps	Sample collection & phenotype assessment activities	Group 1 Control N=14	Group 2 Dexamethasone-treated N=13
**Day 0 & 1 at LA (24hr cycle; day&night)**			
1	Questionnaire, Day 0 at LA, *Morning*	✓	✓
2	ECHO, Day 0 at LA, *Morning*	✓	✓
3	Blood draw, Day 0 at LA, *Morning*	✓	✓
4	Dexamethasone or Acetazolamide, Day 1 at LA, *Morning*	×	✓
5	Dexamethasone or Acetazolamide, Day 1 at LA, *Evening*	×	✓
6	Blood draw, Day 1 at LA, *Evening*	✓	✓
7	Rest at Sealand, overnight	rest	Rest
**Day 2 (day 1) starts at HA**			
	Fly/induction to HA, Day 1 at HA, *Morning*	rest	Rest
3	Dexamethasone or Acetazolamide, Day 1 at HA, *Morning*	×	✓
5	Blood Draw, Day 1 at HA, *Evening*	✓	✓
6	Dexamethasone or Acetazolamide, Day 1 at HA, *Evening*	×	✓
**Day 3 (day 2) continue at HA**			
1	Dexamethasone or Acetazolamide, Day 2 at HA, *Morning*	×	✓
2	Dexamethasone or Acetazolamide, Day 2 at HA, *Evening*	×	✓
**Day 4 (day 3) continue at HA**			
1	Dexamethasone or Acetazolamide, Day 3 at HA, *Morning*	×	✓
2	Dexamethasone or Acetazolamide, Day 3 at HA, *Evening*	×	✓
3	Blood Draw, Day 3 at HA, *Evening*	✓	✓
4	X ray, Day 3 at HA, Evening	✓	✓
5	Echo, Day 3 at HA, Evening	✓	✓
**Day 5 (day 4) continue at HA**			
1	Echo continues, Day 4 at HA, Morning	✓	✓
**Day 6: All three groups fly back to Delhi**			
**Day 13: 7 days after coming back to LA**			
1	Echo, 7 days after coming back to LA	✓	✓

N, number of subjects

Table S2: Baseline clinical characteristics and Baseline Routine Blood chemistry parameters in the Ctrl andDex groups, both at LA and HA.

A. Clinical characteristics		Control Group (n = 14)	Dexamethasone Group (n = 13)	P* Value
1.	Age, years	26.0 ± 1.6	26.0 ± 2	Ns
2.	Female, n	06	06	-
3.	Male, n	08	07	-
4.	Height, cm	165.1 ± 2.1	165.0 ± 3.2	Ns
5.	Weight, Kg	70.2 ± 3.5	65.0 ± 3.6	Ns
6.	BMI, kg/m2	26.0 ± 1.1	24.0 ± 0.7	Ns
7.	SBP, mmHg	116.4 ± 1.7	114.6 ± 1.6	Ns
8.	DBP, mmHg	76.8 ± 1.1	77.5 ± 1.0	Ns
9.	SaO2, %	95.1 ± 0.5	95.2 ± 0.4	Ns
10.	Heart Rate, beats/min	71.1 ± 0.9	71.4 ± 0.9	Ns
11.	Haemoglobin (g/dL)	13.9 ± 1.5	13.5± 1.4	Ns
**B. Routine Blood chemistry**				
1.	Random blood sugar (mg/dL)	93.0 ± 6.2	93.9 ± 9.3	Ns
**Complete Blood count**				
2.	White blood cells (cells/mm^3)	6314.3±1121.1	7100 ±1521.6	Ns
3.	Polymorphs (%)	60.1 ± 9.1	56.6 ± 8.7	Ns
4.	Lymphocytes (%)	31.6 ± 8.3	32.6 ± 9.7	Ns
5.	Monocytes (%)	5.6 ± 1.3	5.9 ± 1.6	Ns
6.	Eosinophils (%)	3.1 ± 2.4	4.4 ± 2.7	Ns
7.	Platelet counts (thousand/mm^3)	155.6 ± 104.8	306.7 ± 269.9	Ns
**Lipid Profile**				
8.	Cholesterol (mg/dL)	152.9 ± 24.6	160.0 ± 25.8	Ns
9.	Triglycerides (mg/dL)	110.8 ± 41.3	116.7 ± 40.8	Ns
10.	High density lipoprotein (mg/dL)	48.1 ± 11.6	41.1 ± 4.8	Ns
11.	Low density lipoprotein (mg/dL)	82.6 ± 23.4	95.6 ± 26	Ns
**Kidney and liver function tests**				
12.	Urea (mg/dL)	23.5 ± 5.2	24.1 ± 6.8	Ns
13.	Creatinine (mg/dL)	0.8 ± 0.2	0.8 ± 0.1	Ns
14.	Sodium (mEq/L)	140.1 ± 2.7	140.5 ± 2.2	Ns
15.	Potassium (mEq/L)	4.6 ± 0.2	4.4 ± 0.2	Ns
16.	Total bilirubin (mg/dL)	0.5 ± 0.2	0.4 ± 0.2	Ns
17.	Total protein (g/dL)	8.0 ± 0.3	7.9 ± 0.3	Ns
18.	Albumin (g/dL)	5.0 ± 0.2	4.8 ± 0.2	Ns
19.	Alkaline phosphatase (U/L)	86.4 ± 10.5	80.5 ± 19.3	Ns
20.	Serum glutamic-oxaloacetic transaminase (U/L)	27.0 ± 13.4	19.9 ± 7.3	Ns
21.	Serum glutamic pyruvic transaminase (U/L)	29.6 ± 14.5	20.6 ± 10.4	Ns

Data are presented as mean ± SEM and are compared by one way ANOVA and unpaired Student's t test. * *P*<0.05 was considered statistically significant; ns, non-significant;. n, number of subjects;BMI, body mass index; SBP, systolic blood pressure; DBP, diastolic blood pressure; SaO_2, arterial oxygen saturation, percent.

Table S3A: SBP in the Ctrl andDex groups, both at LA and HA.

S.No.	Time-point	Ctrl group (n = 14)	Dex group (n = 13)
		SBP (mmHg)	SBP (mmHg)
1.	Day 0 at LA	116.4 ± 6.4	114.6 ± 5.8
2.	Day 1 at LA	117.2 ± 12.1	121.0 ± 14.3
3.	Day 1 at HA	128.0 ± 11.3	122.1 ± 12.9
4.	Day 2 at HA	125.9 ± 10.2	123.5 ± 10.9
5.	Day 3 at HA	127.5 ± 13.0	120.8 ± 15.0

P value comparisons w.r.t SBP within and between the groups

S.No.	Time-point	P values Ctrl gp vs day 0 at LA	P values Dex gp vs day 0 at LA	P values Ctrl vs Dex gp
1.	Day 0 at LA	-	-	Ns
2.	Day 1 at LA	ns	Ns	Ns
3.	Day 1 at HA	ns	Ns	Ns
4.	Day 2 at HA	ns	Ns	Ns
5.	Day 3 at HA	*	Ns	Ns

Data are presented as mean ± SD and are compared by unpaired Student's t test. n, number of subjects;SBP, systolic blood pressure; Ctrl, Control; Dex, dexamethasone; LA, low altitude; HA, high-altitude; gp, group; vs versus * P<0.05 was considered statistically significant; ns, non-significant.

Table S3B: SBP in Females (Pink) and Males (blue) in the Ctrl, and Dex groups, both at LA and HA.

S No.	Time point	Ctrl group (n = 6)	Dex group (n = 6)
		SBP (mmHg)	SBP (mmHg)
1.	Day 0 at LA	113.7 ± 8.3	111.0 ± 3.2
2.	Day 1 at LA	110.7 ± 9.2	112.2 ± 9.8
3.	Day 1 at HA	121.8 ± 12.1	112.8 ± 8.6
4.	Day 2 at HA	125.0 ± 12.3	117.2 ± 9.6
5.	Day 3 at HA	124.3 ± 12.6	112.3 ± 12.5
**S. No.**	**Time point**	**Ctrl group (n = 8)**	**Dex group (n = 7)**
		**SBP (mmHg)**	**SBP (mmHg)**
1.	Day 0 at LA	118.5 ± 3.2	117.7 ± 5.9
2.	Day 1 at LA	122.2 ± 11.7	128.7 ± 13.2
3.	Day 1 at HA	133.4 ± 7.1	130.1 ± 10.6
4.	Day 2 at HA	126.5 ± 8.6	129.8 ± 8.1
5.	Day 3 at HA	129.8 ± 12.9	129.3 ± 12.3

P value comparisons w.r.t SBP within and between the groups

S No.	Time point	P values Ctrl gp vs day 0 at LA	P values Dex gp vs day 0 at LA	P values Ctrl vs Dex gp
1.	Day 0 at LA	-	-	Ns
2.	Day 1 at LA	ns	ns	Ns
3.	Day 1 at HA	ns	ns	Ns
4.	Day 2 at HA	ns	ns	Ns
5.	Day 3 at HA	ns	ns	Ns
**S. No.**	**Time point**	**P values Ctrl gp vs day 0 at LA**	**P values Dex gp vs day 0 at LA**	**P values Ctrl vs Dex gp**
1.	Day 0 at LA	-	-	Ns
2.	Day 1 at LA	ns	Ns	Ns
3.	Day 1 at HA	*	Ns	Ns
4.	Day 2 at HA	ns	Ns	Ns
5.	Day 3 at HA	ns	Ns	Ns

Data are presented as mean ± SD and are compared by unpaired Student's t test. n, number of subjects; SBP, systolic blood pressure; Ctrl, Control; Dex, dexamethasone; LA, low altitude; HA, high-altitude; gp, group; vs versus * P<0.05 was considered statistically significant; ns, non-significant.

Table S4A: DBP in the Ctrl and Dex groups, both at LA and HA.

S.No.	Time point	Ctrl group (n = 14)	Dex group (n = 13)
		DBP (mmHg)	DBP (mmHg)
1.	Day 0 at LA	76.8 ± 4.1	77.5 ± 3.6
2.	Day 1 at LA	78.8 ± 9.4	75.6 ± 10.9
3.	Day 1 at HA	91.3 ± 7.8	81.0 ± 9.4
4.	Day 2 at HA	89.3 ± 7.3	79.6 ± 9.0
5.	Day 3 at HA	89.8 ± 6.9	79.5 ± 11.4

P value comparisons w.r.t DBP within and between the groups

S.No.	Time point	P values Ctrl gp vs day 0 at LA	P values Dex gp vs day 0 at LA	P values Ctrl vs Dex gp
1.	Day 0 at LA	-	-	Ns
2.	Day 1 at LA	ns	Ns	Ns
3.	Day 1 at HA	***	Ns	#
4.	Day 2 at HA	***	Ns	#
5.	Day 3 at HA	***	Ns	#

Data are presented as mean ± SD and are compared by unpaired Student's t test. n, number of subjects; DBP, diastolic blood pressure; Ctrl, Control; Dex, dexamethasone; LA, low altitude; HA, high-altitude; gp, group; vs versus */# *P*<0.05 was considered statistically significant; ns, non-significant.

Table S4B: DBP in Females (Pink) and Males (blue) in the Ctrl and Dex groups, both at LA and HA.

S. No.	Time point	Ctrl group (n = 6)	Dex group (n = 6)
1.	Day 0 at LA	73.4 ± 2.5	77.0 ± 4.2
2.	Day 1 at LA	80.1 ± 11.5	75.8 ± 15.7
3.	Day 1 at HA	91.0 ± 8.6	81.0 ± 7.1
4.	Day 2 at HA	88.8 ± 8.7	77.1 ± 4.7
5.	Day 3 at HA	89.4 ± 5.9	75.6 ± 10.4
**S. No.**	**Time point**	**Ctrl group (n = 8)**	**Dex group (n = 7)**
1.	Day 0 at LA	80.2 ± 1.4	78.0 ± 2.8
2.	Day 1 at LA	77.5 ± 7.3	75.5 ± 3.1
3.	Day 1 at HA	93.1 ± 6.6	81.1 ± 11.0
4.	Day 2 at HA	89.9 ± 6.3	82.0 ± 11.3
5.	Day 3 at HA	90.4 ± 7.6	83.3 ± 11.1

P value comparisons w.r.t DBP within and between the groups

S. No.	Time point	P values Ctrl gp vs day 0 at LA	P values Dex gp vs day 0 at LA	P values Ctrl vs Dex gp
1.	Day 0 at LA	-	-	ns
2.	Day 1 at LA	ns	ns	ns
3.	Day 1 at HA	*	ns	ns
4.	Day 2 at HA	ns	ns	#
5.	Day 3 at HA	*	ns	#
**S. No.**	**Time point**	**P values Ctrl gp vs day 0 at LA**	**P values Dex gp vs day 0 at LA**	**P values Ctrl vs Dex gp**
1.	Day 0 at LA	-	-	ns
2.	Day 1 at LA	-	Ns	Ns
3.	Day 1 at HA	**	Ns	#
4.	Day 2 at HA	*	Ns	Ns
5.	Day 3 at HA	*	Ns	Ns

Data are presented as mean ± SD and are compared by unpaired Student's t test. n, number of subjects; DBP, diastolic blood pressure; Ctrl, Control; Dex, dexamethasone; LA, low altitude; HA, high-altitude; gp, group; vs versus. * P<0.05 was considered statistically significant; ns, non-significant.

Table S5A: HR in the Ctrl and Dex groups, both at LA and HA.

S.No.	Time point	Ctrl group (n = 14)	Dex group (n = 13)
1.	Day 0 at LA	71.1 ± 3.4	71.4 ± 3.3
2.	Day 1 at LA	71.7 ± 3.0	72.5 ± 4.4
3.	Day 1 at HA	98.3 ± 15.2	85.0 ± 15.4
4.	Day 2 at HA	104.0 ± 15.8	88.2 ± 12.8
5.	Day 3 at HA	101.4 ± 13	87.3 ± 15.9

P value comparisons w.r.t HR within and between the groups

S.No.	Time point	P values Ctrl gp vs day 0 at LA	P values Dex gp vs day 0 at LA	P values Ctrl vs Dex gp
1.	Day 0 at LA	-	-	Ns
2.	Day 1 at LA	ns	Ns	Ns
3.	Day 1 at HA	***	Ns	Ns
4.	Day 2 at HA	***	***	#
5.	Day 3 at HA	***	***	##

Data are presented as mean ± SD and are compared by unpaired Student's t test. n, number of subjects; HR, heart rate; Ctrl, Control; Dex, dexamethasone;; LA, low altitude; HA, high-altitude; gp, group; vs versus. * $P<0.05$ was considered statistically significant; ns, non-significant.

Table S5B: HR in Females (Pink) and Males (blue) in the Ctrl and Dex groups, both at LA and HA.

S. No.	Time point	Ctrl group (n = 6)	Dex group (n = 6)
1.	Day 0 at LA	72.0 ± 4.6	71.6 ± 3.3
2.	Day 1 at LA	72.2 ± 4.0	70.7 ± 5.1
3.	Day 1 at HA	103.5 ± 17.6	82.5 ± 13.0
4.	Day 2 at HA	114.8 ± 11.6	87.8 ± 3.0
5.	Day 3 at HA	106.3 ± 13.9	90.6 ± 18.3
**S. No.**	**Time point**	**Ctrl group (n = 8)**	**Dex group (n = 7)**
1.	Day 0 at LA	70.2 ± 0.7	71.1 ± 3.0
2.	Day 1 at LA	71.3 ± 1.1	74.4 ± 2.9
3.	Day 1 at HA	93.0 ± 8.1	87.1 ± 16.9
4.	Day 2 at HA	97.2 ± 13.8	88.5 ± 17.9
5.	Day 3 at HA	97.6 ± 10.3	84.0 ± 12.1

P value comparisons w.r.t HR within and between the groups

S. No.	Time point	P values Ctrl gp vs day 0 at LA	P values Dex gp vs day 0 at LA	P values Ctrl vs Dex gp
1.	Day 0 at LA	-	-	Ns
2.	Day 1 at LA	ns	Ns	Ns
3.	Day 1 at HA	***	Ns	Ns
4.	Day 2 at HA	***	**	##
5.	Day 3 at HA	***	Ns	Ns
**S. No.**	**Time point**	**P values Ctrl gp vs day 0 at LA**	**P values Dex gp vs day 0 at LA**	**P values Ctrl vs Dex gp**
1.	Day 0 at LA	-	-	Ns
2.	Day 1 at LA	ns	Ns	#
3.	Day 1 at HA	***	Ns	Ns
4.	Day 2 at HA	***	Ns	Ns
5.	Day 3 at HA	***	Ns	#

Data are presented as mean ± SD and are compared by unpaired Student's t test. n, number of subjects; HR, heart rate; Ctrl, Control; Dex, dexamethasone;; LA, low altitude; HA, high-altitude; gp, group; vs versus. * $P<0.05$ was considered statistically significant; ns, non-significant

n

Table S6: Echocardiogram parameters, Inferior vena cava diameter and collapsibility in the Ctrl and dexamethasone groups at LA (Pre-induction to HA), HA and back at LA (Upon return from HA).

Parameters	Time point	Ctrl group	P Value	Dex group	P Value	P Value
**A. IVC Diameter**		**cm**		**cm**		
**Both Sexes**		**(n = 14)**		**(n = 13)**		
	LA (Pre-induction to HA)	0.8 ± 0.3	-	0.8 ± 0.1	-	-
	HA	1.6 ± 0.3	≤0.001	1.4 ± 0.3	≤0.001	0.033
	LA (Upon return from HA)	0.8 ± 0.3		0.8 ± 0.2		
**Females**		**(n = 6)**		**(n = 6)**		-
	LA (Pre-induction to HA)	0.9 ± 0.4	-	0.8 ± 0.1	-	-
	HA	1.6 ± 0.3	0.006	1.4 ± 0.0	0.003	-
	LA (Upon return from HA)	0.9 ± 0.4		0.7 ± 0.1		-
		**(n = 8)**		**(n = 7)**		-
**Males**	LA (Pre-induction to HA)	0.8 ± 0.2	-	0.8 ± 0.2	-	-
	HA	1.6 ± 0.2	≤0.001	1.5 ± 0.3	0.001	-
	LA (Upon return from HA)	0.8 ± 0.1		0.8 ± 0.2		-

**B. IVC Collapsibility**						
		**(n = 14)**		**(n = 13)**		
**Both Sexes**	LA (Pre-induction to HA)	collapsible 8 subjects		Collapsible 9 subjects		
	HA	collapsible		collapsible		
	LA (Upon return from HA)	collapsible		Collapsible		
		**(n = 6)**		**(n = 6)**		
**Females**	LA (Pre-induction to HA)	collapsible 2 subjects		Collapsible 4 subjects		
	HA	collapsible		collapsible		
	LA (Upon return from HA)	collapsible		Collapsible		
**Males**		**(n = 8)**		**(n = 7)**		
	LA (Pre-induction to HA)	collapsible 6 subjects		Collapsible 5 subjects		
	HA	collapsible		collapsible		
	LA (Upon return from HA)	collapsible		Collapsible		

Data are presented as mean ± SD. n, number of subjects; IVC, Inferior vena cava; Ctrl, Control; Dex, dexamethasone; LA, low altitude; HA, high-altitude. * P<0.05 was considered statistically significant; ns, non-significant.

Table S7: Echocardiogram parameters, RV and LV systolic function, LV, LA, RA size, IVS(d/s), PWD(d/s) in Ctrl, and Dex groups at LA (Pre-induction to HA), HA and back at LA (Upon return from HA).

	Parameters	Time point	Ctrl group (n = 14)	Dex group (n = 13)
A.	RV function (TAPSE) (mm)	LA (Pre-induction to HA)	21.1 ± 1.2	21.0 ± 0.2
		HA	21.2 ± 1.8	21.4 ± 0.6
		LA (Upon return from HA)	21.1 ± 1.0	21.1 ± 0.3
B.	LV function (%) (LVEF %)	LA (Pre-induction to HA)	65.5 ± 2.0	64.9 ± 1.6
		HA	62.0 ± 0.0	63.0 ± 0.0
		LA (Upon return from HA)	64.5 ± 1.8	65.5 ± 2.3
C.	LV size (cm)	LA (Pre-induction to HA)	4.5 ± 0.4 / 2.8 ± 0.3	4.3 ± 0.4 / 2.7 ± 0.3
		HA	4.4 ± 0.2 / 2.4 ± 0.2	4.4 ± 0.2 / 2.3 ± 0.1
		LA (Upon return from HA)	4.4 ± 0.5 / 2.9 ± 0.3	4.2 ± 0.4 / 2.7 ± 0.2
D.	LA size (cm)	LA (Pre-induction to HA)	2.7 ± 0.3	2.5 ± 0.4
		HA	2.8 ± 0.3	2.6 ± 0.2
		LA (Upon return from HA)	2.8 ± 0.3	2.5 ± 0.3
E.	RA size (cm)	LA (Pre-induction to HA)	3.3 ± 0.3	3.3 ± 0.2
		HA	3.3 ± 0.3	3.3 ± 0.2
		LA (Upon return from HA)	3.4± 0.3	3.3 ± 0.2
F.	IVS(d/s) (cm)	LA (Pre-induction to HA)	1.0 ± 0.1 / 1.2 ± 0.1	0.9 ± 0.1 / 1.2 ± 0.1
		HA	0.9 ± 0.1 / 1.2 ± 0.1	0.9 ± 0.0 / 1.2 ± 0.1
		LA (Upon return from HA)	0.9 ± 0.1 / 1.2 ± 0.1	0.9 ± 0.1 / 1.1 ± 0.1
G.	PWD(d/s) (cm)	LA (Pre-induction to HA)	1.1 ± 0.2 / 1.3 ± 0.2	1.0 ± 0.1 / 1.3 ± 0.1
		HA	0.9 ± 0.1 / 1.2 ± 0.1	0.9 ± 0.0 / 1.2 ± 0.1
		LA (Upon return from HA)	1.0 ± 0.1 / 1.2 ± 0.1	0.9 ± 0.1 / 1.2 ± 0.1

Data are presented as mean ± SEM and are compared by one way ANOVA and unpaired Student's t test. n, number of subjects; RV, right ventricle; TAPSE, Tricuspid annular plane systolic excursion; LV, left ventricle; LVEF, LV ejection fraction; LA, left auricle; RA, right auricle; IVS(d/s), Interventricular septum dimension in end diastole and end systole; PWD(d/s), Posterior wall in end diastole and end systole; Ctrl, Control; Dex, dexamethasone;; LA, low altitude; HA, high-altitude.

Table S8A: SaO_2 in the Ctrl andDex groups, both at LA and HA.

S.No.	Time point	Ctrl group (n = 14)	Dex group (n = 13)
1.	Day 0 at LA	-	-
2.	Day 1 at LA	95.1 ± 2.1	95.2 ± 1.5
3.	Day 1 at HA	86.7 ± 4.2	89.0 ± 2.3
4.	Day 2 at HA	-	-
5.	Day 3 at HA	89.7 ± 5.2	90.9 ± 2

P value comparisons w.r.t SaO_2 within and between the groups

S.No.	Time point	P values Ctrl gp vs day 1 at LA	P values Dex gp vs day 1 at LA	P values Ctrl vs Dex gp
1.	Day 0 at LA	-	-	-
2.	Day 1 at LA	-	-	ns
3.	Day 1 at HA	***	***	ns
4.	Day 2 at HA	-	-	-
5.	Day 3 at HA	***	***	ns

Data are presented as mean ± SD and are compared by unpaired Student's t test. n, number of subjects; SaO_2, arterial oxygen saturation, percent; Ctrl, Control; Dex, dexamethasone;; LA, low altitude; HA, high-altitude; gp, group; vs versus. * *P*<0.05 was considered statistically significant; ns, non-significant

Table S8B: SaO_2 in Females (Pink) and Males (blue) in the Ctrl, and Dex groups, both at LA and HA.

S. No.	Time point	Ctrl group (n = 6)	Dex group (n = 6)
1.	Day 0 at LA	-	-
2.	Day 1 at LA	95.3 ± 1.2	94.6 ± 1.8
3.	Day 1 at HA	87.0 ± 3.0	89.1 ± 2.5
4.	Day 2 at HA	-	-
5.	Day 3 at HA	90.5 ± 3.7	90.3 ± 2.4
**S. No.**	**Time point**	**Ctrl group (n = 8)**	**Dex group (n = 7)**
1.	Day 0 at LA	-	-
2.	Day 1 at LA	94.8 ± 2.4	95.5 ± 0.9
3.	Day 1 at HA	86.3 ± 4.8	88.8 ± 1.8
4.	Day 2 at HA	-	-
5.	Day 3 at HA	89.1 ± 5.6	91.5 ± 0.7

P value comparisons w.r.t SaO_2 within and between the groups

S. No.	Time point	P values Ctrl gp vs day 1 at LA	P values Dex gp vs day 1 at LA	P values Ctrl vs Dex gp
1.	Day 0 at LA	-	-	-
2.	Day 1 at LA	-	-	Ns
3.	Day 1 at HA	***	**	Ns
4.	Day 2 at HA	-	-	-
5.	Day 3 at HA	*	*	Ns
**S. No.**	**Time point**	**P values Ctrl gp vs day 1 at LA**	**P values Dex gp vs day 1 at LA**	**P values Ctrl vs Dex gp**
1.	Day 0 at LA	-	-	-
2.	Day 1 at LA	-	-	Ns
3.	Day 1 at HA	*	***	Ns
4.	Day 2 at HA	-	-	-
5.	Day 3 at HA	ns	Ns	Ns

Data are presented as mean ± SD and are compared by unpaired Student's t test. n, number of subjects; SaO_2, arterial oxygen saturation, percent; Ctrl, Control; Dex, dexamethasone; LA, low altitude; HA, high-altitude; gp, group; vs versus. * *P*<0.05 was considered statistically significant; ns, non-significant.
